# Integrating Workplace Learning Into Healthcare Settings: A Mixed‐Methods Study to Inform Curriculum Design, Resource Allocation and Organisational Support

**DOI:** 10.1155/jonm/3021423

**Published:** 2026-06-28

**Authors:** Yuhan Ho, Doreen Heng, Wentao Zhou, Emily Neo Kim Ang, Yah Shih Chan, Chiew-Jiat Rosalind Siah

**Affiliations:** ^1^ Alice Lee Centre for Nursing Studies, Yong Loo Lin School of Medicine, National University of Singapore, Singapore, Singapore, nus.edu.sg; ^2^ Alexandra Hospital, Singapore, Singapore, ah.com.sg

**Keywords:** competence assessment, leadership support, mixed methods, organisational support, postgraduate nursing education, resource allocation, workplace learning

## Abstract

**Introduction:**

Postgraduate education is critical for advancing nursing competence, yet enrolment has declined as nurses struggle to balance clinical, academic and personal responsibilities. Workplace learning offers a flexible, context‐driven alternative, allowing nurses to integrate academic development into their clinical practice and potentially mitigating work–life conflicts. However, adoption of structured workplace learning remains limited across healthcare organisations.

**Aim:**

This study aimed to explore how workplace learning can be integrated into healthcare settings to inform curriculum design, resource allocation and organisational support in postgraduate nursing education.

**Design and Methods:**

An explanatory sequential mixed‐methods study comprising a single‐cohort quantitative pre–post knowledge assessment and repeated competence assessments, followed by qualitative focus groups to explain implementation enablers and barriers. Twenty‐three postgraduate nurses enrolled in a 13‐week workplace learning course participated; 22 completed the study. Quantitative data were analysed with nonparametric statistics, while qualitative data underwent thematic analysis.

**Results:**

We observed a slight improvement in median knowledge scores and increasing competence scores across four time points. As this was a single‐group evaluation without a control group, quantitative findings cannot be interpreted as causal effects. Qualitative analysis generated three themes: (1) learning and assessment, (2) integration of workplace learning in the organisation and (3) organisational impact on workplace learning.

**Conclusion:**

Workplace learning was feasible and valued when supported by peer learning structures, appropriately timed assessments and organisational enablers such as protected time, adequate infrastructure and leadership support.

## 1. Introduction

Postgraduate education for nurses, defined as any higher education pursued after obtaining a bachelor’s degree, plays a critical role in advancing the nursing profession [1–3]. Notably, it is closely associated with increased knowledge and competence for effective interprofessional collaboration and linked to improved patient outcomes and higher standards of professional practice [4, 5]. Despite its importance, recent years have seen a decline in postgraduate nursing enrolment with many nurses citing the challenge of balancing demanding clinical roles with academic pressures, as well as family commitments, and financial burdens, as significant barriers to further education [6–9].

To overcome the challenges faced by many nurses in pursuing further education, one of the most well‐established and effective pedagogical approaches is workplace learning, which provides a flexible and pragmatic alternative to traditional classroom‐based postgraduate education for nurses to learn within their actual clinical environments. This approach also supports nurses in maintaining a healthy work–life balance, since it reduces the need for time away from the workplace and allows learning to be integrated seamlessly into daily professional responsibilities [10, 11]. This model aligns with core principles of adult learning theory, or andragogy, which posits that adult learners are self‐directed, bring a wealth of experience to their education and are motivated by learning that is immediately relevant and applicable to their professional problems. This perspective is complemented by Kolb’s experiential learning theory, which explains the process through which workplace learning offers nurses the opportunity to turn clinical experiences into meaningful and lasting knowledge to enhance their competence and is vital for promoting teamwork and collaboration among healthcare professionals [12, 13].

By learning in the context of actual healthcare settings, nurses will have the opportunity to strengthen their interprofessional communication and collaborative skills which are key competencies for delivering safe and effective patient care [14, 15]. Through structured, supervised workplace learning experiences, nurses can receive immediate feedback, reflect on their practice and engage in meaningful interactions that enhance both personal competence and the collective performance of healthcare teams.

Despite the well‐recognised theoretical and practical advantages of workplace learning, such as improved clinical competence, enhanced teamwork and more seamless translation of knowledge to practice, its widespread adoption in healthcare settings remains limited. Recent studies indicate that only a modest proportion of health organisations globally have systematically integrated workplace learning into their professional development frameworks. For instance, a 2022 scoping review found that structured workplace‐based learning initiatives are actively implemented in fewer than 30% of healthcare organisations worldwide, with significant regional variation [16]. This aligns with findings from a multinational survey by Berkhout et al. [17], which revealed that while over 80% of healthcare professionals acknowledge the value of workplace learning, only approximately 25% report regular involvement in structured workplace learning programmes within their organisations.

The underutilisation of workplace learning is attributed to various barriers, including organisational constraints, insufficient organisational support and a lack of standardised frameworks for implementation and evaluation [16, 18]. When not implemented effectively, this gap in professional development can contribute to negative management indicators such as staff burnout, turnover and compromised patient safety. Given these challenges, there is a pressing need for further high‐quality evidence to elucidate the most effective strategies for promoting and sustaining workplace learning in diverse healthcare contexts. This evidence is critical for informing policy, guiding organisational investment and ultimately enhancing patient care outcomes through continual workforce development.

Despite recognition of workplace learning as an effective pedagogical approach, there remains limited empirical guidance on how to operationalise workplace learning within healthcare organisations in ways that align curriculum structure with resource allocation, leadership support, protected time and role boundary management for practising nurses. This gap is particularly relevant for nursing management because implementation requires organisational decisions on rostering, infrastructure, facilitator capacity and competing service demands.

## 2. Aims

The primary aim of this study is to explore how workplace learning can be integrated into healthcare settings to inform curriculum design, resource allocation and organisational support in postgraduate nursing education.

Questions:

1. What were the changes in participants’ knowledge scores (pre/post) and competence ratings (across four time points) during the course?
2. What were participants’ experiences of the enablers and barriers to implementing workplace learning in the healthcare organisation?

## 3. Methods

### 3.1. Study Design

An explanatory sequential mixed‐methods design was used. First, quantitative data were collected from a single cohort using a pre–post knowledge assessment and repeated competence assessments at four time points to describe outcome trends across the course. Second, qualitative focus group discussions were conducted after course completion to explore participants’ experiences and to explain organisational and educational factors that may underpin the observed quantitative trends. Integration occurred at two points: (i) quantitative findings informed refinement of the qualitative interview guide (connecting) and (ii) findings were integrated during interpretation through narrative weaving across strands.

### 3.2. Setting, Participants and Sampling Method

Participants were recruited via pragmatic purposive sampling from a single cohort of postgraduate nurses enrolled in a 13‐week workplace learning course on integrated healthcare delivered through collaboration between a university and a partnering healthcare organisation. The sample size was determined by total course enrolment (*N* = 23); no a priori sample size calculation was conducted. Eligibility criteria were: (1) postgraduate nurses aged ≥ 21 years; (2) able to speak and write in English and (3) full‐time registered nurses. Participants who were unable to complete the course were excluded from analysis.

### 3.3. Intervention

**FIGURE 1:**
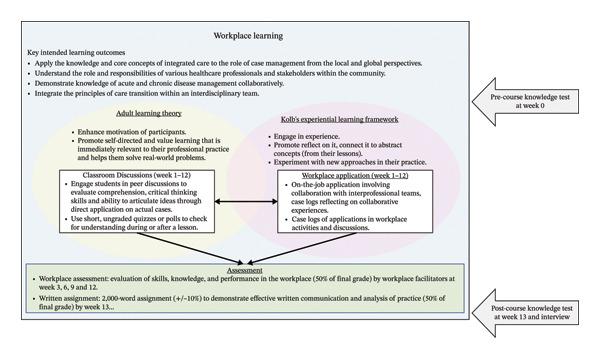
Framework of curriculum sequence, learning objectives and the standardisation of assessment criteria in workplace learning.

The 13‐week course was co‐designed by a university and a partnering healthcare organisation (Figure 1). Curriculum development was guided by workplace learning pedagogy, which prioritises learning through authentic experiences and supports reflection on practice within real healthcare settings. This approach was intended to ensure the educational content was relevant and immediately applicable to participants’ professional roles.

Each week, participants attended a 2‐h lesson focusing on key concepts and skills related to integrated healthcare with an emphasis on community care. Following each lesson, they engaged in structured workplace application activities, enabling them to apply learning in simulated or actual work environments across the 13 weeks. This combination of theoretical instruction and practice‐based application aimed to reinforce learning and develop competence in essential nursing practices.

To evaluate learning and provide feedback, experienced facilitators from the healthcare organisation conducted competency assessments at four time points over the course. Sessions followed a standard weekly format (2‐h lesson followed by workplace application), and any deviations due to operational constraints were documented by the course team for reflection and iterative improvement. The assessments were intended to monitor progress, identify areas for improvement and provide timely feedback. To support consistent delivery and assessment, facilitators were briefed on course objectives and the competence assessment criteria prior to implementation.

### 3.4. Outcome Measures

A knowledge assessment related to integrated healthcare and community‐related topics comprised 40 multiple‐choice items and was administered before (pre‐test) and after (post‐test) the course. Items were developed to align with course learning objectives to assess the intended knowledge domains. The maximum attainable score was 40, with higher scores indicating higher knowledge. The knowledge questionnaire was developed by a content expert to align with course learning objectives; formal psychometric validation was not conducted. Facilitators received orientation on the scoring rubric to promote consistent use across time points.

The Clinical Assessment Tool for Competence comprised six items measured on a 9‐point Likert scale, ranging from 1 (Need Improvement) to 9 (Exceed Expectation). Items were communication, professionalism, patient care, clinical practice, clinical knowledge/reasoning and safe practice. The tool for postgraduate nurses was adapted from a simulation‐based assessment tool for undergraduate nursing students, with a content validity index of 0.97 and an intraclass correlation coefficient of 0.799–0.929 [19]. In this study, Cronbach’s alpha was 0.94, indicating high internal consistency.

A semistructured interview guide was developed to address the study research questions and was informed by the quantitative findings. One pilot session was conducted to check clarity and flow of questions.

### 3.5. Data Collection

At the beginning of the course, eligible individuals were invited to participate. Those who agreed provided written informed consent prior to data collection. During a course session, all consenting participants completed the 40‐item knowledge questionnaire to assess baseline knowledge related to the course content (Figure 1).

From 14 August 2024 to 13 November 2024, participants were assessed at four time points spaced 3 weeks apart. Competence was assessed using the Clinical Assessment Tool by facilitators, to evaluate participants’ clinical competencies and progression over the course. Upon course completion, participants completed the same 40‐item knowledge questionnaire as the post‐test to describe any change in knowledge over the course period.

After course completion, participants were invited to focus group discussions to explore experiences of workplace learning implementation, including enablers, barriers and organisational supports required. Qualitative sampling was bounded by the enrolled cohort. A pilot focus group was conducted by a female researcher with doctoral‐level training to ensure questions aligned with study objectives and were appropriate for eliciting relevant information; minor refinements were made thereafter. We monitored thematic saturation across groups by reviewing the evolving codebook after each session; saturation was operationalised as the point at which no substantively new codes, perspectives or implementation issues were identified in the final group, and this judgement was confirmed through team discussion.

In total, three focus groups were conducted (*n* = 7, 7 and 8), lasting 42–61 min. Discussions were audio‐ and video‐recorded and transcribed verbatim for analysis. The semistructured guide was refined using quantitative findings to probe explanations for observed outcome trends. For instance, given that the quantitative data showed competence scores were lowest at week 3, the interview guide included specific prompts asking participants to elaborate on their experience with the timing of early assessments. Similarly, questions were added to explore potential reasons behind the only slight improvement in knowledge scores, despite the clear progression in competence ratings. Participants were reminded to maintain confidentiality of information shared during the focus groups. Field notes were taken to document contextual observations and group dynamics.

### 3.6. Data Analysis

Quantitative data were analysed using Jamovi version 2.3.28 (The Jamovi Project, 2024). Descriptive statistics were used for sociodemographic variables and presented as frequency distributions. Mean (M) and standard deviation (SD) were used for continuous variables. Statistical significance was set at *p* < 0.05 (two‐sided) with a 95% confidence interval. Given the small sample size (*n* = 22), nonparametric tests were used [20]. Median and interquartile range (IQR) were used to summarise knowledge and competence scores. Differences between pre‐ and post‐test knowledge scores were analysed using the Wilcoxon signed‐rank test. Differences in competence scores across four time points were analysed using Friedman’s test followed by the Durbin–Conover post hoc test. In addition to *p* values, effect sizes were computed to support interpretation of practical significance.

Focus group discussions were transcribed verbatim, and participants were assigned identification numbers (e.g., P1) to protect confidentiality. Transcripts were managed in Microsoft Excel (Microsoft Corporation, 2024). Qualitative data were analysed using a codebook approach to thematic analysis. This approach was chosen as it is well suited to team‐based research, where developing a structured codebook enhances consistency and rigour across multiple analysts [21]. Two researchers familiarised themselves with the transcripts and generated initial codes. An initial codebook was developed and iteratively refined through discussion, with differences resolved through consensus. Codes were organised into candidate themes and reviewed against the dataset to ensure coherence and adequate coverage. An audit trail documenting codebook revisions and analytic decisions was maintained. Themes are presented with supporting quotations.

Quantitative findings were used to refine the qualitative interview guide (connecting). Findings were integrated during interpretation using narrative weaving, whereby quantitative trends (e.g., assessment timing concerns, competence progression) were examined alongside qualitative themes to generate implementation‐focused meta‐inferences relevant to curriculum design, resource allocation and organisational support.

### 3.7. Rigour

Trustworthiness was supported through (i) credibility: analyst triangulation with consensus meetings and examination of deviant cases; (ii) dependability: maintenance of an audit trail documenting coding and codebook revisions; (iii) confirmability: reflexive notes and transparent linkage of interpretations to verbatim excerpts and (iv) transferability: detailed description of the setting, participants and course structure [22].

### 3.8. Ethical Considerations

Approval from the university’s Institutional Review Board was obtained prior to study commencement (IRB‐2024‐369). Participants were informed of the study purpose and provided with information sheets. Voluntary participation was emphasised, and written consent was obtained prior to data collection. Participants were assured that study participation would not influence their course grades.

## 4. Results

**FIGURE 2:**
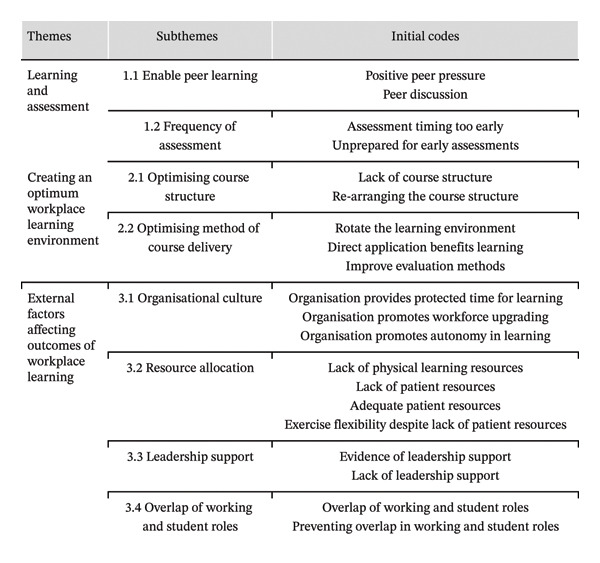
Coding table of qualitative data.

Twenty‐three postgraduate nurses met the eligibility criteria and consented to participate. One participant did not complete the course due to medical reasons and was excluded from analysis, leaving 22 participants. The mean age was 35.64 years (SD = 6.31), and most participants were female (86.4%). Years of working experience ranged from 1 to 21 years (*M* = 9.95, SD = 6.51), and 20 participants (90.0%) held a bachelor’s degree (Table 1). Three focus groups were conducted (*n* = 7, 7 and 8), lasting 42–61 min. Analysis generated three main themes and eight subthemes describing influences on participants’ knowledge and competence during workplace learning (Figure 2).

**TABLE 1:** Participant characteristics.

Characteristics	Number of participants (*n* = 22)
	Mean (SD)/*n* (%)
Age	35.64 (6.31)
Range	26–46
Gender	
Male	3 (13.6)
Female	19 (86.4)
Ethnicity	
Chinese	11 (50.0)
Malay	5 (22.7)
Indian	1 (4.5)
Others	5 (22.7)
Highest academic qualification	
Diploma	2 (9.1)
Bachelor’s degree	20 (90.9)
SNB post‐registration programmes	
Advanced diploma	4 (18.2)
Current work designation	
Staff nurse	7 (31.8)
Senior staff nurse	11 (50.0)
Assistant nurse clinician	4 (18.2)
Years of work experience	
Mean (SD)	9.95 (6.51)
Range	1–21
Attended similar workplace learning course	
Yes	1 (4.5)
No	21 (95.5)

Abbreviation: SD, Standard Deviation.

### 4.1. Knowledge and Competence Level

Median knowledge scores had a slight increased from 36.5 (IQR = 4.0) to 37.0 (IQR = 3.0) between the pre‐test and post‐test knowledge questionnaires. Competence scores increased across the four time points, from 38.0 (IQR = 12.5) at week 3 to 45.0 (IQR = 4.0) at week 12 (*X* ^2^ = 45.86, *p* < 0.001) (Table 2). An analysis of effect sizes was conducted to determine the magnitude of the observed changes. The knowledge scores from pre‐test to post‐test corresponded to an effect size of *r* = 0.39, which is considered a medium effect, and the competence scores across the four time points yielded a Kendall’s W of 0.70, indicating a large effect size. As this evaluation used a single‐group design without a comparator, the observed changes should be interpreted as within‐cohort trends and cannot be attributed causally to the workplace learning course. Table 3 presents a joint display that connects these numerical results to the explanatory themes derived from participant experiences.

**TABLE 2:** Quantitative results knowledge and competence levels.

**Knowledge questionnaire scores**	**Pre-test**	**Post-test**	**W**		**p** **value**	
	**Median (IQR)**					

	36.5 (4.0)	37.0 (3.0)	47.00		0.03	

**Clinical assessment tool scores**	**Week 3**	**Week 6**	**Week 9**	**Week 12**	**X** ^2^	**p** **value**
	**Median (IQR)**					

	38.0 (12.5)	38.0 (12.8)	39.5 (9.0)	45.0 (4.0)	45.86	< 0.001

**Test statistics (** **p** **value)**	**Week 3**	**Week 6**	**Week 9**	**Week 12**		

Week 3	—	2.79 (0.007)	6.56 (< 0.001)	11.29 (< 0.001)	—	—
Week 6	—	—	3.76 (< 0.001)	8.49 (< 0.001)	—	—
Week 9	—	—	—	4.73 (< 0.001)	—	—

*Note:* IQR = interquartile range, *X* ^2^ = chi‐square, *p* value = 0.05; W = Wilson signed rank test.

**TABLE 3:** Joint display integrating quantitative and qualitative findings.

Quantitative finding	Qualitative explanation and meta‐inference	Illustrative quote(s)
Finding 1: Competence scores increased progressively with a large effect size (Kendall’s *W* = 0.70), but scores were lowest at the first assessment point in week 3.	This quantitative trend is explained by participants feeling unprepared for early assessments, which they felt were premature and suppressed their initial scores. The subsequent increase in competence is explained by the power of peer learning, where sharing diverse clinical experiences and observing colleagues helped build confidence and practical skills over the duration of the course. Relevant Themes: Theme 1: Learning and assessment ‐ *Subtheme 1: Enable Peer Learning* ‐ *Subtheme 2: Frequency of Assessment*	‘I feel like having it [assessment] on the 3rd and 6th week is a little bit too early, for me ah at least’ (FGD 2_P13). ‘See what other people are doing and from there you can, like, pickup on what to improve’ (FGD 2_P13).
Finding 2: Knowledge scores showed a slight but significant improvement with a medium effect size (*r* = 0.39) from pre‐test to post‐test.	The modest quantitative gain is illuminated by the qualitative data, which shows that the true value for participants was in the immediate, practical application of theoretical knowledge. Learning was most effective when concepts from lessons could be directly applied in the clinical setting, reinforcing retention and making the knowledge ‘fresh’. Peer discussions also contributed by exposing participants to unfamiliar cases. Relevant Themes: Theme 1: Learning and assessment ‐ *Subtheme 1: Enable Peer Learning* Theme 2: Integration of workplace learning ‐ *Subtheme 2: Optimising Method of Course Delivery*	‘So I find it (direct application) very beneficial in the sense that the information are all still fresh in my mind’ (FGD 2_P15). ‘We learn new things also…we never encounter that kind of case before so yea, we learn new things’ (FGD 2_P9).
Finding 3: (Meta‐inference from overall results) The feasibility and individual success of the workplace learning program were highly dependent on the organisational context.	This meta‐inference explains the variability in the learning experience. While the course provided the educational structure, its day‐to‐day effectiveness was moderated by organisational factors. Participants’ ability to engage was either enabled by supportive structures or hindered by their absence. This highlights that successful implementation is contingent on organisational readiness. Relevant Themes: Theme 3: Organisation impact on workplace learning ‐ *Subtheme 1: Organisational Culture* (e.g., protected time) ‐ *Subtheme 2: Resource Allocation* (e.g., room access) ‐ *Subtheme 4: Overlap of Working and Student Roles*	‘Supportive in a sense that they give us TL, protected time’ (FGD 1_P3). ‘So because uh our challenge is for us, we need to find a room, empty room to interview patient. But sometimes maybe all the room occupied’ (FGD 1_P7). ‘We have to set boundaries. Because otherwise… uh you cannot concentrate on your work’ (FGD 2_P10).

### 4.2. Theme 1: Learning and Assessment

These findings suggest that learning and assessment in workplace settings benefit significantly from structured peer learning and appropriately timed evaluations. Peer engagement was found to be effective during the workplace learning in enabling nurses to bridge knowledge gaps, adapt theory to practice and build professional confidence. At the same time, aligning assessment timing with the natural progression of clinical learning ensures more accurate measurement of competence. These insights can inform curriculum design by embedding intentional opportunities for collaboration and reflection, as well as by scheduling assessments in a way that supports nurses’ realistic development trajectories.

#### 4.2.1. Subtheme 1: Enable Peer Learning

Participants valued the opportunity for peer discussion, which facilitated the exchange of diverse clinical experiences and exposure to unfamiliar cases. This peer interaction enabled participants to identify knowledge gaps, reflect on their own practice and enhance their learning. ‘*We learn new things also. Yea for example, because I’m from inpatient ward right then that time (P14) was presenting [one case]…we never encounter that kind of case before so yea, we learn new things’ (FGD 2_P9).*

Peer learning also fostered a collaborative environment, allowing nurses to adapt theoretical concepts to practical situations and highlighting the importance of structured peer engagement in curriculum design. ‘*See what other people are doing and from there you can, like, pickup on what to improve’ (FGD 2_P13).*

#### 4.2.2. Subtheme 2: Frequency of Assessment

Participants reported that assessments conducted in weeks 3 and 6 were premature, with many feeling unprepared to demonstrate competence so early in the course. ‘*I feel like having it [assessment) on the 3rd and 6th week is a little bit too early, for me ah at least’ (FGD 2_P13).* This suggests the need for curriculum planners to align assessment schedules with natural learning progression, introducing formative assessments early and reserving summative assessments for later stages after adequate clinical exposure.

### 4.3. Theme 2: Integration of Workplace Learning in a Healthcare Organisation

The integration of workplace learning within healthcare organisations is strongly influenced by course structure and delivery methods. Early and clear introduction of foundational skills builds learner confidence, while varied and timely opportunities for practical application enhance knowledge retention and adaptability. These findings advocate for a curriculum that is both well‐sequenced and flexible, promoting contextual learning through rotations and hands‐on activities. Such an approach not only supports the immediate learning needs of nurses but also generates evidence for ongoing refinement of educational strategies and resource deployment.

#### 4.3.1. Subtheme 1: Optimising Course Structure

Initial confusion regarding course organisation was common, particularly during the first few lessons. The preference for foundational content and key frameworks to be introduced earlier in the course underscores the importance of course scaffolding. ‘The OLDCART comes later, which could come in the front to enhance our interview skills’ (FGD 1_P3). Early delivery of core modules supports orientation, reduces anxiety and provides an evidence base for sequencing curriculum content to bridge theory and practice.

#### 4.3.2. Subtheme 2: Optimising Method of Course Delivery

Direct, immediate application of new knowledge in clinical settings was widely appreciated for aiding retention and contextual understanding. Participants also valued being rotated to different clinical environments, which broadened their perspective and adaptability. ‘*There’s really a beauty of like, learning from different discipline…they have different perspective on how to do things and how to handle patients so it’s really good’ (FGD 3_P16).*

Suggestions included incorporating more diverse activities, such as case scenarios, to facilitate contextual learning. ‘*So I find it (direct application) very beneficial in the sense that the information are all still fresh in my mind’ (FGD 2_P15).* These preferences inform curriculum design by highlighting the benefits of experiential, practice‐based learning and cross‐setting exposure.

### 4.4. Theme 3: Organisation Impact on Workplace Learning

Organisational factors play a pivotal role in the success of workplace learning. A culture that values learning, allocates resources equitably and provides strong leadership support enables nurses to focus on professional growth without undermining their work–life balance. Addressing challenges such as resource limitations and role conflict is crucial for creating optimal learning environments. These findings underscore the importance of ongoing organisational commitment and responsive resource planning to safeguard the effectiveness and sustainability of workplace learning initiatives.

#### 4.4.1. Subtheme 1: Organisational Culture

A supportive organisational culture was instrumental in promoting workplace learning. The provision of protected learning time, encouragement for professional advancement and scheduling flexibility enabled nurses to engage deeply with the curriculum without compromising their work–life balance. ‘*Supportive in a sense that they give us TL, protected time’ (FGD 1_P3).* These factors highlight the role of organisational policies in resource allocation and professional development.

#### 4.4.2. Subtheme 2: Resource Allocation

Resource availability varied, with some participants able to access sufficient cases and support, while others faced challenges like securing interview rooms or computers. ‘*So because uh our challenge is for us, we need to find a room, empty room to interview patient. But sometimes maybe all the room occupied’ (FGD 1_P7).* These challenges could sometimes be mitigated by creative solutions, such as using electronic health records when patients were unavailable. This points to the need for healthcare organisations to regularly assess and address resource gaps to ensure equitable and effective workplace learning.

#### 4.4.3. Subtheme 3: Leadership Support

Leadership, especially in the form of flexible rostering and supervisor support, was vital to participants’ ability to balance work and study. ‘*I just want to affirm my, my RO. Because they gave me an option. So now I’m working morning shift for the whole year because I’m studying’ (FGD 2_P11).* While support was generally appreciated, inconsistent consideration of study commitments in roster planning sometimes hindered learning. This highlights the importance of leadership engagement and communication in supporting nurses’ professional growth.

#### 4.4.4. Subtheme 4: Overlap of Working and Student Roles

Participants often experienced role conflicts, with work‐related interruptions during learning sessions. Some addressed this by using visual indicators (like training badge) to delineate their learner status. ‘*We have to set boundaries. Because otherwise… uh you cannot concentrate on your work. SO it’s a good thing that our uniform itself there is no partial blue long. So the patient themselves will not find you’ (FGD 2_P10).* These findings underscore the need for role clarity and clear boundaries, both in practice and policy, to optimise focus during learning sessions.

## 5. Discussion

This study provides insights into how workplace learning may be integrated in a healthcare context to inform curriculum design, resource allocation and organisational support, with the aim of strengthening alignment between educational activities and the practical realities of nurses’ work. The findings can be interpreted through the lens of adult learning theory, which underscores the importance of learner autonomy, experience and practical relevance in professional education.

Peer learning emerged as a key feature of workplace learning, enabling nurses from different clinical backgrounds to exchange practice‐based insights, identify knowledge gaps and build confidence. Participants described how peer discussion helped them recognise areas for improvement, consistent with evidence that peer learning can support deeper understanding and collaborative problem‐solving [23, 24]. Embedding structured peer learning activities (e.g., case discussions and peer feedback) may help develop context‐specific knowledge and skills that are applicable to clinical practice [25].

A supportive organisational culture, characterised by leadership encouragement, flexible scheduling and protected learning time, was also important for participation in workplace learning [11, 26]. These findings resonate with adult learning theory, which emphasises that adults learn best when they can immediately apply new knowledge to solve real‐world problems. The value participants placed on direct application of concepts and peer discussions (Themes 1 and 2) highlights their motivation as experienced professionals to engage in learning that is contextually relevant and practical.

These findings can also be interpreted through the Job Demands‐Resources (JD‐R) model. The challenges participants faced, such as role overlap and interruptions, function as ‘job demands’ that can lead to burnout. Conversely, enablers like peer learning and leadership support operate as critical ‘job resources’ that help employees achieve goals and reduce demands. These resources also include tangible provisions; for example, adequate allocation of patient cases, private spaces and technology reduces barriers to engagement. Where such resource constraints were present, participants described workarounds to sustain learning, highlighting the critical need for organisations to formalise supportive policies and regularly review resource availability. By framing the findings this way, it becomes clear that successful workplace learning requires organisations to actively cultivate both cultural and material resources to buffer the inherent demands of balancing work and study.

Managing overlap between clinical and learner roles is important for optimising workplace learning, as role conflict may disrupt engagement and contribute to stress [10, 18]. From an adult learning perspective, this role conflict is a critical barrier, as adult learners are simultaneously managing professional, academic and personal responsibilities. Participants described interruptions from routine duties during designated learning periods, suggesting that clearer role boundaries and protected time may support participation. Organisational policies that designate and protect learning time can signal institutional commitment and support professional development without displacing clinical responsibilities [11, 18].

Participants’ accounts also suggest structural tensions typical of implementing workplace learning within service‐driven environments. Difficulty accessing interview rooms, computers or suitable cases reflects a mismatch between organisational expectations for training and the infrastructure required to enact it. Constrained resources may amplify role conflict when nurses are expected to meet clinical demands while fulfilling learner responsibilities within the same shift. Conversely, protected time and supportive rostering appeared to facilitate engagement, reflection and application of learning, indicating that organisational supports function as enabling conditions for sustained workplace learning rather than optional ‘add‐ons’.

From a nursing management perspective, sustainability depends on whether workplace learning is embedded within workforce planning rather than layered onto existing workload. Key considerations include staffing backfill for protected learning time, availability of physical space and devices, and facilitator capacity. Without planned resourcing, workplace learning may become an additional burden that undermines participation over time. Future evaluations should therefore examine longer‐term organisational outcomes (e.g., retention intentions, unit‐level workflow disruption and perceived return‐on‐investment) alongside learner outcomes.

### 5.1. Limitations

This study has limitations. The quantitative component involved a small single cohort without a control group; changes in knowledge and competence ratings may reflect maturation, familiarity with assessment expectations or assessor‐related effects rather than intervention effects. The knowledge questionnaire was developed by a content expert and was not psychometrically validated, limiting interpretation of score changes. Furthermore, while the Clinical Assessment Tool for Competence has strong reported psychometric properties from its original validation (Tan et al., 2016), inter‐rater reliability was not reassessed among the facilitators in this specific study, which should be considered when interpreting the competence scores. The qualitative findings are based on focus group discussions, meaning the insights were co‐constructed through group interaction and reflect the collective experience of the participants. As the study was conducted in a single programme and organisational context, the findings offer a deep, context‐specific understanding rather than broad generalisability.

### 5.2. Clinical Implications

Despite these limitations, the findings suggest that workplace learning can be feasible for postgraduate nurses when supported by organisational enablers. For implementation, organisations should formalise protected time for workplace learning within roster planning so that learning is not treated as additional work. Practical steps include (i) rostering that ring‐fences learning blocks, (ii) ensuring access to minimum infrastructure (quiet rooms, computers, EHR access), (iii) clarifying learner‐versus‐clinician role boundaries during training periods and (iv) identifying and resourcing facilitators to provide feedback and conduct assessments consistently. These measures align resource allocation with curriculum requirements and may improve feasibility for postgraduate nurses balancing service and study. While these findings highlight the necessity of formalising resource allocation, this study was not designed to determine the most effective operational model and thus cannot provide evidence to recommend specific strategies, such as adjusted staffing ratios versus overtime.

### 5.3. Implications for Nursing Education

This study suggests that workplace learning may be a viable approach for postgraduate nursing education by supporting application of learning within clinical settings. Direct application of concepts in practice and structured peer learning opportunities were described as helpful for learning and competence development. Future workplace learning curricula could incorporate these approaches, alongside continued collaboration between healthcare organisations and educational institutions to strengthen organisational support. Consideration of resourcing and leadership support remains important when designing workplace learning programmes.

### 5.4. Recommendations for Future Research

Longitudinal research is recommended to examine retention of knowledge and skills and the sustainability of workplace learning. Future studies could include larger samples across multiple organisations to improve transferability. Research examining downstream outcomes for patients and organisations (e.g., care processes, workforce outcomes and operational indicators) would further clarify the potential benefits of workplace learning beyond learner outcomes. Specifically, research is needed to compare the effectiveness, feasibility and cost‐benefit of different staffing models (e.g., backfill, adjusted ratios) for creating protected learning time in busy clinical environments.

## 6. Conclusion

This explanatory sequential mixed‐methods study described outcome trends and explored implementation experiences of workplace learning for postgraduate nurses. We observed small improvements in knowledge scores and increasing competence ratings over the course, alongside qualitative evidence that peer learning opportunities, appropriate assessment timing and well‐sequenced delivery supported learning. Organisational enablers, particularly protected time, adequate infrastructure, leadership support and clear role boundaries, were pivotal for feasibility. Future multisite studies with comparative designs and validated measures are needed to evaluate effectiveness and sustainability.

## Funding

This research received no commercial or not specific grant from any funding agency in the public, commercial or not‐for‐profit sectors.

## Conflicts of Interest

The authors declare no conflicts of interest.

## Data Availability

The data that support the findings of this study are available upon request from the corresponding author. The data are not publicly available due to privacy or ethical restrictions.
